# Health Benefits of Oily Fish: Illustrated with Blue Shark (*Prionace glauca*), Shortfin Mako Shark (*Isurus oxyrinchus*), and Swordfish (*Xiphias gladius*)

**DOI:** 10.3390/nu15234919

**Published:** 2023-11-25

**Authors:** Franklin Chamorro, Paz Otero, Maria Carpena, Maria Fraga-Corral, Javier Echave, Sepidar Seyyedi-Mansour, Lucia Cassani, Miguel A. Prieto

**Affiliations:** Nutrition and Bromatology Group, Department of Analytical Chemistry and Food Science, Instituto de Agroecoloxía e Alimentación (IAA)—CITEXVI, Universidade de Vigo, 36310 Vigo, Spain; franklin.noel.chamorro@uvigo.es (F.C.); paz.otero@uvigo.es (P.O.); mcarpena@uvigo.es (M.C.); mfraga@uvigo.es (M.F.-C.); javier.echave@uvigo.es (J.E.); sepidar.seyyedi@uvigo.es (S.S.-M.); luciavictoria.cassani@uvigo.es (L.C.)

**Keywords:** oily fish, polyunsaturated fatty acids, fish consumption, human health, risk-benefit ratio

## Abstract

Oily fish is a rich source of energy, proteins, essential amino acids, lipids, vitamins, and minerals. Among the macronutrients with the highest contribution are lipids, mainly long-chain omega 3 polyunsaturated fatty acids (ω-3 LC-PUFA), especially eicosapentaenoic acid (EPA) and docosahexaenoic acid (DHA). Both EPA and DHA play a beneficial role in promoting health and preventing many diseases, including cardiovascular diseases, such as stroke and acute myocardial infarction. They also contribute to the prevention of neurological, metabolic, and immune-system-related diseases, as well as supporting body-weight control. Oily fish consumption is also important at different stages of human life, from conception to old age. For example, DHA plays an important role in brain and retina development during fetal development and in the first two years of life, as it positively influences neurodevelopment, such as visual acuity, and cognitive functions. In contrast with the possible health benefits of the intake of oily fish, the presence of certain chemical pollutants, for example, heavy metals, can be a risk for the health of consumers, mainly in sensitive population groups such as pregnant women and children under 2 years of age. The presence of these pollutants is influenced to a greater extent by fish species, their role in the trophic chain, and their size. However, various studies state that the benefits outweigh the risk of consuming certain species. This review will be focused on the health benefits of the intake of three oily fish species, namely blue shark (*Prionace glauca*), shortfin mako shark (*Isurus oxyrinchus*), and swordfish (*Xiphias gladius*).

## 1. Introduction

Over the past two decades, consumer concerns related to food safety and quality issues have increased worldwide. To meet the growing expectation of high-quality food, holistic analytical approaches have been developed to comprehensively assess the potential risks and benefits associated with food consumption [1]. Many studies have focused on the benefits of consuming different kinds of seafood, including oily fish. Highly migratory species (HMS) listed by the United Nations Convention on the Law of the Sea (UNCLOS) in Article 64 include different species of oily fish highly captured and consumed in Europe, such as blue shark (*Prionace glauca)*, shortfin mako shark *(Isurus oxyrinchus)*, and swordfish (*Xiphias gladius)* [2]. In 2021, about 69,000 tons of these species were fished, which accounted for up to 13% of the total earnings of the Spanish fleet [3]. The nutritional characteristics of these oily fish have turned them into highly commercialized species, so they have economic significance [4].

Blue shark (*P. glauca*) is an elasmobranch species of the Carcharhinidae family, with an elongated body and large eyes, and an average length of 2.5 m and weight that can reach up to 80 kg. It is noteworthy that *P. glauca* is significantly more prominent in the North Atlantic than in the Pacific and Indian Oceans. It is a marine predator and is common and abundant in oceanic fisheries, mainly feeding on fish such as mackerel, herring, grouper, horse mackerel, bonito, gadidae, squid, and seabirds. According to some studies, dietary patterns differ between sexes and age categories [4,5]. 

The shortfin mako (*I. oxyrinchus*), present in all temperate and tropical seas, is a large, fast-swimming, and migratory shark species, an elasmobranch in the Lamnidae family. *I. oxyrinchus* also feed on fast-moving teleost fish such as tuna, bluefish, and billfish. Several physiological adaptations can be observed in this species, including ridged dermal denticles, internalized red muscle, large gill slits, a torpedo-shaped body, lunate caudal fins, and thunniform swimming mode, which have emerged through evolutionary adaptation [6]. 

The swordfish (*X. gladius*) is a pelagic fish from tropical and temperate waters. This species is a unique member of the Xiphiidae family, which lives in shallow waters between 200 and 600 m deep and at temperatures between 18 and 22 °C. They are large, highly migratory predatory fish distinguished by their long, flattened beaks. The Atlantic, Pacific, and Indian Oceans, as well as the Mediterranean Sea, are fisheries for this species [7]. 

These three species of oily fish are highly rich in macro- and micro-nutrients, including proteins, essential amino acids, lipids, vitamins, and minerals. It is well known that oily fish are the primary dietary source of the long-chain omega 3 polyunsaturated fatty acids (ω-3 LC-PUFA), particularly eicosapentaenoic (EPA) and docosahexaenoic acid (DHA) [8]. Both EPA and DHA play a beneficial role in the health promotion and prevention of many diseases, such as cardiovascular diseases (CVD). The effect of these PUFAs may alleviate the risk of stroke, acute myocardial infarction, and hypertension, and they may help with brain development in childhood. In addition, EPA plus DHA have useful effects on neurological and metabolic diseases, body weight control, and immunological system-related diseases [9].

As opposed to the potential health benefits of dietary fish intake, its frequent consumption has raised concerns regarding exposure to several chemical pollutants, such as mercury (Hg), which becomes concentrated in the muscles of most species, mainly in methylmercury (MeHg). Globally, fish consumption and dietary exposure to Hg are highly correlated due to the different amounts and variety of fish consumed, as well as the cultural traditions associated with its consumption. Large and long-lived predatory species such as sharks, swordfish, and large tuna have been reported to contain high concentrations of MeHg, which are positively correlated with the species’ age, weight, or size [10]. Human overdose of organic or inorganic Hg has been described to produce diverse adverse effects on the central nervous and cardiovascular systems, both in humans and animals [8]. Moreover, MeHg intake by mothers has been related to significant health consequences associated with IQ decline in children [11]. In this regard, the World Health Organization (WHO) and the Food and Agriculture Organization (FAO) have established a provisional tolerable weekly intake (PTWI) for MeHg of 1.3 µg/kg body weight [12]. In addition, the recommendation for some sections of the population, including children; very frequent fish consumers; and pregnant, nursing, and childbearing-age women, is to limit or avoid the consumption of large fish recognized to contain MeHg. However, the exposure data and risks associated with Hg and MeHg in fish vary. In this context, several studies have concluded that the consumption of certain fish species possess a serious health risk [10,13,14]. Meanwhile, some other studies have provided contradictory reports where the risk-benefit assessments showed that the frequency of consumption of some seafood to increase the benefits of EPA plus DHA could be raised without adverse effects from Hg. Following this trend, additional studies have been conducted to analyze how Hg toxicity is affected by the interaction with selenium (Se). Selenium is an essential trace element that has an antioxidant and anticancer potential, and may offer protection by counteracting the toxicity of MeHg [15]. 

As a result of these potentially conflicting effects, the public has become increasingly confused about both the risks and benefits of fish consumption in recent years. Despite this, research suggests that moderate consumption of these species may benefit consumer health rather than threaten it. Their high nutritional values may counteract the negative impacts of heavy metal exposure. Therefore, this review aims to discuss the health benefits from the intake of three oily fish species, namely blue shark (*P. glauca*), shortfin mako shark (*I. oxyrinchus*), and swordfish (*X. gladius*), and to evaluate the risk-benefit associated with the consumption of these selected species.

## 2. Nutritional Composition

**Figure 1:**
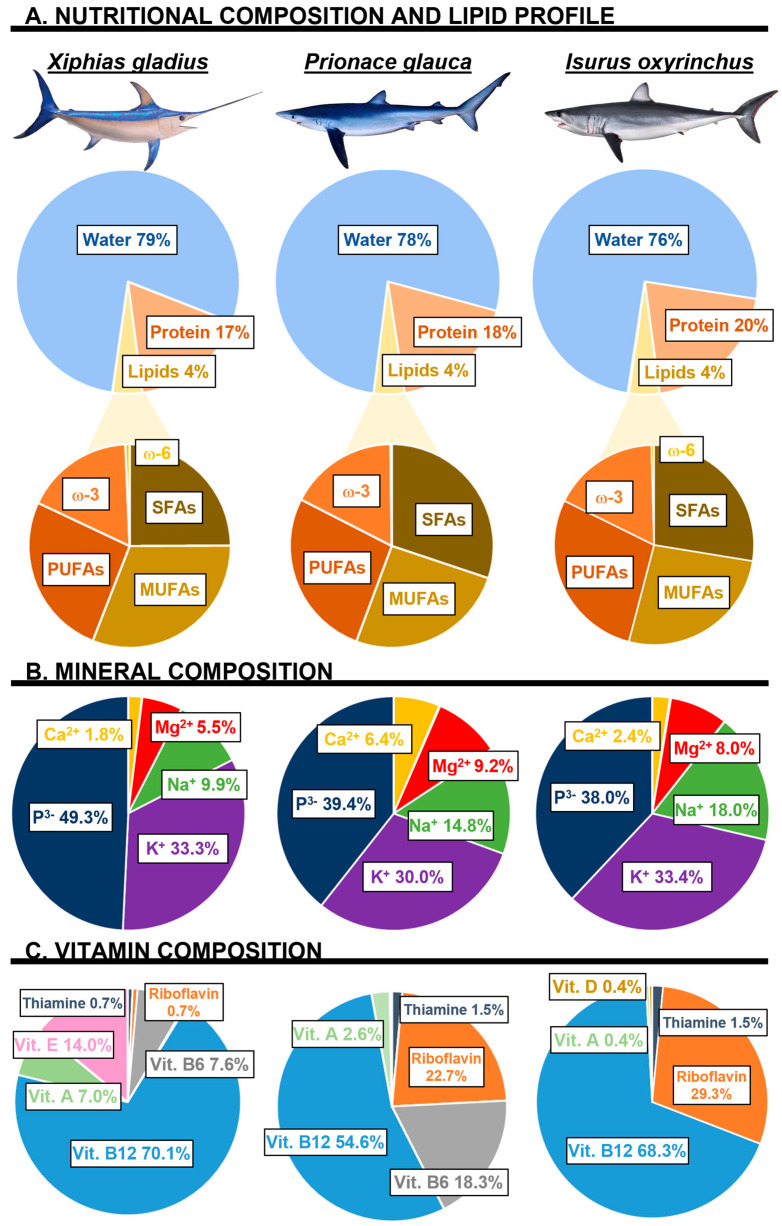
Nutritional composition of the species *Xiphias gladius*, *Prionace glauca*, and *Isurus oxyrinchus* expressed in percentage (%). (**A**) Nutritional composition and lipid profile. (**B**) Mineral composition. (**C**) Vitamin composition. *Note*: those pie charts that do not reach 100%, correspond to small percentages (0.1–0.2%) of (**B**) other minerals (Fe, I, Zn) and (**C**) vitamins (folates, vitamin D).

Fish products play a valuable role in human nutrition; approximately 20% of the protein of animal origin ingested worldwide comes from fish and shellfish [16]. Fish are mainly made up of water (52 to 82%), proteins (16 to 21%), carbohydrates (approximately 0.5%), lipids (0.5 to 2.3%), vitamins, and minerals, with the protein and lipid fractions highlighted as major components [17]. However, the nutritional composition of fish can vary depending on the species, environmental factors, age, sex, state of maturation, migratory behavior, source of feeding, and even within each species [18]. In this sense, various databases provide information about the approximate composition of fish and shellfish. Among these are the global database of FAO International Network of Food Data Systems (INFOODS), the United States Department of Agriculture (USDA), the National Marine Fisheries Service of the United States (NOAA), and the Department of Health and Social Care of the United Kingdom (DHSC). In addition, several authors have analyzed the nutritional composition of various species of fish and shellfish [18,19,20,21]. The innumerable nutritional benefits of fish consumption are known, due to its contribution of proteins with a high biological value; ω-3 PUFAs; low cholesterol levels; rich content of fat-soluble vitamins, such as vitamins A and D; and high contribution of essential minerals [22,23]. In this section, the nutritional composition of blue fish is highlighted, paying attention to the species *P. glauca I. oxyrinchus*, and *X. gladius*, as reflected in Figure 1.

### 2.1. Moisture Content

The moisture content in fish can vary between 60 and 80%, depending on the species. It is known that the water content is inversely proportional to the fat content, and that the sum of both is around 80%. In this way, fish with 65–70% humidity indicates that it has a high-fat content. In addition, the interaction of water content with other components, such as proteins and fats, influences the texture of fish [24]. In this sense, the moisture content of the three species ranged between 76 and 78.7% (Table 1), highlighting *X. gladius*. These data confirm that all three are classified as oily fish due to their high concentration of lipids [25,26].

### 2.2. Protein and Amino Acid Profile 

The protein content of fish varies between 17 to 23% depending on the species, stage of maturation, stages of starvation, habitat (freshwater, saltwater, or brackish water), and depth; but, unlike lipids, it is not influenced by the time of year [17]. In this sense, the protein content of these species ranges between 17 and 20.7 g of protein per 100 g of product, *I. oxyrinchus* is the species that provides the most protein (Table 1). These three species stand out for their protein content compared with other oily fish species such as mackerel, trout, eel, and red mullet [18]. As similarly reported by Mesa et al. (2021), who studied the nutritional importance of the fresh fish, shrimp, and mollusks most consumed in Spain, the consumption of 100 g of these species could contribute 50% of the reference daily intake (RDI) value in the human diet [21]. In 2012, The Spanish Ministry of Agriculture, Food, and Environment published guidelines regarding the nutritional qualities of products from extractive fishing and aquaculture to inform consumers about the nutritional and health properties of food products. Furthermore, the Spanish guide for the nutritional qualities of products from extractive fishing and aquaculture, using binomial risk-benefit, maintains that these species have a high protein content [27]. Moreover, this guide establishes that these proteins contribute to increasing or conserving muscle mass and maintaining bones in normal conditions. Additionally, it has been described that fish proteins may provide anti-inflammatory effects due to their ability to reduce pro-inflammatory cytokines (TNF-α, IL-6) and limit the accumulation of pro-inflammatory macrophages at the site of injury [9,28]. On the other hand, experimental studies have reported different health benefits from consuming proteins and protein derivatives from fish, such as the hypocholesterolemic effect. Although the mechanism has not yet been elucidated, it seems that the amino acid composition of fish is responsible [29]. Fish proteins seem to possess antihypertensive effects due to the presence of angiotensin-converting enzyme inhibitor peptides [30,31] and antiatherosclerotic effects [32]. These proteins can improve insulin sensitivity and contribute to the prevention of metabolic syndrome and reduce the risk of type 2 diabetes [32].

The nutritional value of a protein depends on the composition and quantity of essential amino acids and their susceptibility to being digested [33,34,35]. According to the protein digestibility corrected amino acid score (PDCAAS) method, which is used to assess the quality of a protein [33,34,35], the species contain proteins of an excellent quality, contributing close to 100% of the protein score, amino acids, and a PDCAAS of 94% [34]. They are also considered to be of a better quality than red meat due to their lower collagen content and better proteolytic digestion [9,36]. *X. gladius* shows a high content of essential amino acids, namely histidine, isoleucine, leucine, lysine, threonine, tryptophan, valine, phenylalanine, and methionine, providing between 40 and 60% RDI for all of the essential amino acids (Table 1) [37,38,39,40]. This amino acid profile provides *X. gladius* with many health benefits. It has been reported that an adequate supply of histidine through diet has positive effects on neurodegenerative and cognitive disorders related to age, metabolic syndrome, rheumatoid arthritis, and arthritis or inflammatory diseases such as bowel disease [41]. In addition, the contribution of amino acids, such as leucine, isoleucine, and valine, contribute to the regulation of energy homeostasis, metabolism, and innate and adaptive immunity, also participating in glucose metabolism and the synthesis of lipids and proteins [42]. Additionally, phenylalanine and tryptophan are considered natural antidepressants and contribute to proper functioning of the brain [43].

**Table 1:** Nutritional composition of the species *Xiphias gladius*, *Prionace glauca*, and *Isurus oxyrinchus* and energy and nutrient recommendations [9,44]. All data are expressed in g/100 g, except when specified differently.

	*Xiphias gladius*	*Prionace glauca*	*Isurus oxyrinchus*	Reference Daily Intake	Nutritional Declaration
Energy (Kcal)	107	82	87		
Protein	17	18.7	20.7	50	High content
Tryptophan ^†^	0.222	-	-	0.41	
Threonine ^†^	0.868	-	-	1.63	
Isoleucine ^†^	0.912	-	-	1.55	
Leucine ^†^	1.61	-	-	3.43	
Lysine ^†^	1.82	-	-	3.10	
Methionine ^†^	0.586	-	-	1.55	
Phenylalanine ^†^	0.773	-	-	1.14	
Valine ^†^	1.02	-	-	0.41	
Histidine ^†^	0.583				
Cystine	0.212	-	-	2.69	
Tyrosine	0.668	-	-	1.96	
Arginine	1.18	-	-		
Alanine	1.2	-	-		
Aspartic acid	2.03	-	-		
Glutamic acid	2.96	-	-		
Glycine	0.95	-	-		
Proline	0.7	-	-		
Serine	0.808	-	-		
Total lipids	4.3	4.5	4.4		Low in saturated fat
Saturated FAs	1.15	1.57	1.27		
Monounsaturated FAs	1.43	1.33	1.22		
Polyunsaturated FAs	1.2	1.4	1.3		
ω-3	0.800	0.900	0.795		High content of ω-3-FAs
ω-6	0.031	0.01	0.02		
Cholesterol mg/1000 Kcal	39	51	-		
Ratio AGP/AGS	0.86	0.89	1,02		-
Carbohydrates	0	0	0.21		
Fiber	0	0	-		
Water	78.7	78.5	76		
***Minerals***					
Calcium (mg)	19	34	12	800	-
Iron (mg)	0.9	0.8	0.957	14	-
Iodine (μg)	17.2	0	-	150	-
Magnesium (mg)	57	49	40	375	Font
Zinc (mg)	0.4	0.4	0.358	10	-
Sodium (mg)	102	79	90	≤120	Low content
Potassium (mg)	342	160	167	2000	Font
Phosphorus (mg)	506	210	190	700	High content
Selenium (μg)	48.1	28	28.5	55	High content
***Vitamins***					
Thiamine (mg)	0.05	0.04	0.03	1.1	-
Riboflavin (mg)	0.05	0.62	0.58	1.4	-
Niacin equivalents (mg)	9	2.9	2.1	16	High content
Vitamin B6 (mg)	0.51	0.50	-	1.4	Font
Folates (μg)	15	0	-	200	-
Vitamin B12 (μg)	5	1.49	1.35	2.5	High content
Vitamin C (mg)	0	0	0	80	-
Vitamin A: Eq. Retinol (μg)	500	70	8.36	800	High content
Vitamin D (μg)	7.2	8	8	5	High content
Vitamin E (mg)	1	0	0	12	-

^†^ Refers to the essential amino acids. *Note:* The fatty acid profile of the three species and the amino acid profile of *P. glauca* and *I. oxyrinchus* are not currently available in the literature. (-) refers to not determined.

### 2.3. Lipid Content: Fatty Acids Profile and ω-3/ω-6 Relation

The health benefits from the consumption of fish are mainly associated with the contribution of high-quality lipids and a fraction of PUFAs, such as EPA and DHA [9]. The European Food Safety Authority (EFSA) has suggested a recommended daily intake (RDI) of between 250 and 500 mg of EPA plus DHA for European adults, based on cardiovascular risk considerations [45]. In this sense, oily fish can easily fulfill this RDI. The lipid content of the studied species is very similar, ranging between 4.3 and 4.5 g/100 g, highlighting *I. oxyrinchus* with a lipid contribution of 4.5 g/100 g. These species have a high content of lipids and an adequate contribution of PUFAs compared with other species, such as mullet and horse mackerel [18,21,46]. For this reason, they are classified as fatty species or called oily fish. 

The guidelines published in 2012 by the Spanish Ministry of Agriculture, Food, and Environment established a nutritional declaration for these species indicating a low content of saturated fats and high content of ω-3 fatty acids. PUFAs are described as having properties that can contribute to the normal functioning of the heart and the prevention of CVDs [27]. Regarding the fatty acid profile in Table 1, *P. glauca* and *I. oxyrinchus* displayed saturated fatty acids (SFAs) as their main component, with palmitic acid (C16:0) being the main SFA [21]. Meanwhile, *X. gladius* had a higher proportion of monounsaturated fatty acids (MUFAs), highlighting oleic acid (C18:1 n–9) [21]. As far as PUFAs, the results were very similar, ranging between 1.2 and 1.4 g/100 g, highlighting *P. glauca* as the species that contained the highest amount of PUFAs. Within this group, ω-3 stood out, and its content ranged between 0.795 and 0.900 g/100 g. In this sense, Mesa et al. (2021) [21] reported on the fatty acid profile of various species, such as *P. glauca* and *X. gladius*, considering their significant amounts of ω-3-PUFAs, mainly DHA and EPA, which showed similar quantities to those found in salmon and sardines [21]. It was pointed out that consuming a portion of *P. glauca* (150 g) per week would provide approximately 276 mg/day of ω-3 PUFAs [21]. This intake would contribute to covering the indications of the EFSA panel recommending the consumption of at least 250 mg/day of EPA plus DHA to maintain normal brain function and vision, contributing to a reduction in the risk of mortality from coronary heart disease [47,48]. Additionally, ω-6-PUFAs were present in a lower proportion in all fish species and mainly comprised arachidonic acid (C20:4 ω-6) [21], thus favoring the ω-3/ω-6 ratio. In this sense, Mesa et al. (2021) indicated a ratio of ω-3/ω-6 of 5.7/1 in the case of sharks and 17.4/1 in *X. gladius* [21].

### 2.4. Carbohydrates

The concentration of carbohydrates in fish is usually low, approximately 0.3%. They are frequently found in the skeletal muscle in the form of glycogen and as an integral part of nucleotides. The soluble carbohydrate content varied between 0 and 0.21 g/100 g wet weight for all species [49]. 

### 2.5. Vitamins

Vitamins are essential to maintain key functions in the human body. There are two classes, water-soluble and fat-soluble vitamins, with the greater proportion found in the form of fat-soluble vitamins, which are located mainly in the muscle and livers of fish. The species under study stood out as they contained significant amounts of B complex vitamins, comprising niacin (B3) ranging between 2.1 and 9 mg/100 g, pyridoxine (B6) at 0.50 mg/100 g, and cobalamin (B12) ranging between 1.35 and 5 µg/100 g; *X. gladius* was highlighted for its contribution to these vitamins. The consumption of 100 g of these species provides between 25 and 50% RDI of these vitamins (Table 1). The guidelines published in 2012 by the Spanish Ministry of Agriculture, Food, and Environment established the nutritional benefits as possessing a high content of the aforementioned vitamins [27]. Within the nutritional declarations, the guidelines indicate that the consumption of these vitamins contributes to normal energy metabolism, the correct functioning of the nervous system, as well as heart and psychological functioning. Niacin contributes to maintaining normal conditions for the skin and mucous membranes and reduces fatigue. Pyridoxine and cobalamin contribute to the normal functioning of the immune system, the formation of red blood cells, and the process of cell division [27]. Additionally, these species are rich in fat-soluble vitamins such as vitamins A and D. Their consumption can provide between 25 and 80% RDI; thus, they represent a relevant source of these vitamins. The consumption of fat-soluble vitamins is important because they contribute to normal iron metabolism, immune system functioning, and cell differentiation processes [25]. In the particular case of vitamin D, it participates in the maintenance of normal bones and teeth, the maintenance of normal calcium levels in the blood, and the normal absorption and utilization of calcium and phosphorus [50,51,52].

### 2.6. Minerals

Mineral elements are among the most relevant nutrients acquired from fish as they participate in many biological processes as part of numerous enzymes. They cannot be synthesized by humans, so intake through diet is essential [53]. In this context, the species constitute an excellent food source of minerals. Table 1 reports the contribution of minerals present in 100 g of fish. They stand out for their content of magnesium (Mg) at 40–57 mg/100 g, potassium (K) at 160–342 mg/100 g, phosphorus (P) at 190–506 mg/100 g, and selenium (Se) at 28–55 μg/100 g; *X. gladius* is highlighted as contributing the highest amount of these minerals among the species studied. According to the report published by the Spanish Ministry of Agriculture, Food, and Environment in 2012, these species are presented as a relevant source of these minerals. Regarding health claims, Mg contributes to normal energy metabolism, electrolyte balance, normal muscle and nervous system function, normal protein synthesis, and cell division processes [27,54,55]. On the other hand, Se is attributed as having different benefits to health, among which it contributes to the normal functioning of the immune system, normal functioning of the thyroid, and protection of cells against oxidative damage. Se is part of selenoproteins, which are responsible for biological reactions of the reduction-oxidation type, antioxidant defense, metabolism of the thyroid hormone, and immune responses [56,57]. Various studies have reported that Se can protect against some environmental contaminants, such as Hg, which is present in some fish, even though this point will be discussed later [58,59,60,61,62]. On the other hand, these species have a low contribution of sodium (Na) at 79–100 mg/100 g. The nutritional declaration highlights these species as having a low Na content, with their consumption thus being attractive in low-sodium diets, for example, for patients with hypertension. Eating a variety of nutritious foods is the best way to maintain health and prevent chronic disease. As fish is a source of minerals, it should be a main component of a balanced diet. The essentiality of these elements is beyond dispute, and their daily requirements must be fully covered in a healthy diet.

## 3. Health Benefits of Blue Fish: Direct Consumption and By-Product Applications

**Figure 2:**
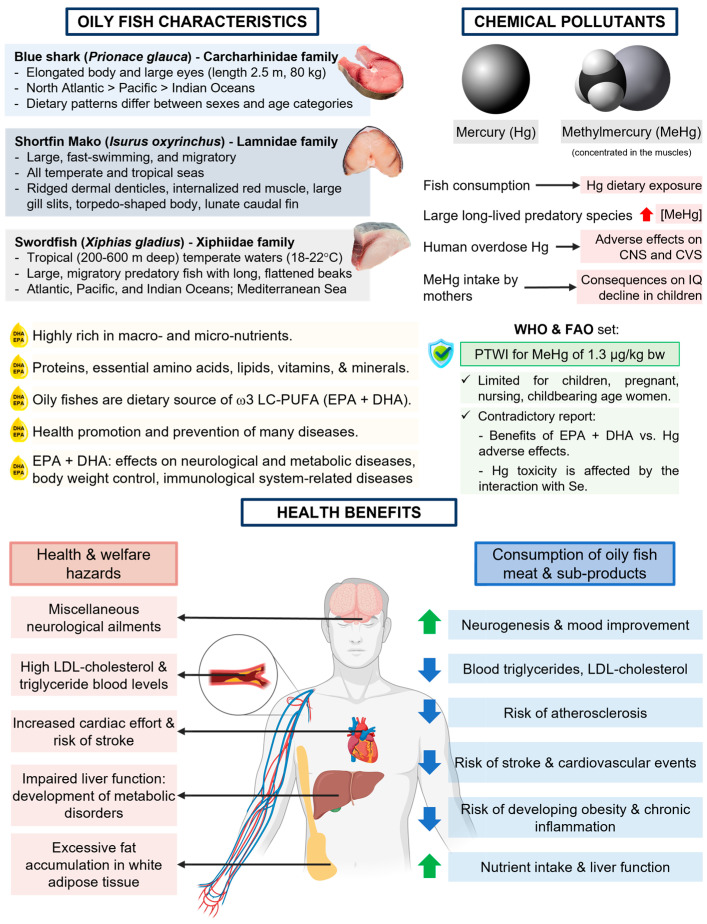
Summary of oily fish characteristics, chemical pollutants, and potential health benefits from the consumption of oily fish.

Considering their nutritional composition, several health benefits have been attributed to the consumption of oily fish, such as cardioprotection, neuroprotection, and the improvement of metabolic functions (Figure 2). These benefits are mainly associated with their high content of w-3 LC-PUFAs, such as DHA, EPA, and docosapentaenoic acid (DPA), as these species are a major natural source; therefore, their consumption, both as fish and oil, can trigger health benefits. These species are also recognized for their high content of minerals, as well as proteins and essential amino acids, which are characterized by their high bioavailability, which contributes to their nutritional and metabolic benefits. Several research works have evaluated the increased consumption of these oily fish through in vivo assays with animal models, clinical trials, and metanalyses. These reports have associated this intake with the health benefits mentioned, even though not all of the mechanisms have been elucidated.

### 3.1. Antioxidant and Anti-Inflammatory

In the last decades, advancements in science and technology have resulted in developing alternative or improved treatments to improve the well-being of the increasing global population affected by chronic diseases. Most of these affections are well recognized to be triggered by oxidation processes related to metabolic deregulations, such as autoimmune diseases. Inflammatory bowel disease, which encompass two types of immune activation and inflammation response, affect the gastrointestinal tract. Two chronic diseases are ulcerative colitis, which mainly affects the colon or rectum, and Crohn’s disease, which affects any segment of the gastrointestinal tract. The global prevalence of these two illnesses is increasing (>2.5 million patients in Europe and 1 million in the USA) and results in high healthcare costs (around EUR 5 billion or USD 6 billion). Their complex etiology includes oxidative stress as a triggering factor; thus, antioxidants are potential coadjutants of conventional treatments [63]. Oxidative environments create free radicals (reactive oxygen species, ROS) that may attack key cellular molecules like membrane lipids, proteins, and DNA [64]. The overproduction of these radicals has been established as one of the main inducers of the onset of chronic inflammation, which, in turn, increases the probability of developing other related disorders, such as diabetes, obesity, and even cancer [65]. Indeed, the oxidation of low-density lipoprotein (LDL) circulating cholesterol is a major cause for developing atherosclerosis, thus increasing the risk of any related CVDs [66]. 

In this sense, LC-PUFAs are recognized as effective antioxidant molecules that may exert antioxidant properties through dietary intake [67]. Furthermore, evidence suggests that not only are PUFAs active antioxidant components in oily fish, but Se also acts as an indirect antioxidant as it is involved in the synthesis of several endogenous antioxidants, such as glutathione peroxidase [68]. The recent clinical trial DEPOXIN, conducted in Slovak children (*n* = 60) between 7 and 18 years old, analyzed a variety of biomarkers related to oxidative stress and blood levels of endogenous antioxidants following the treatment of ω-3 PUFAs rich fish oil [69]. The study found that following treatment, the individuals showed increased superoxide dismutase levels, as well as reduced levels of advanced oxidation products and other oxidative stress biomarkers. Considering that these PUFAs are present in generally high concentrations in the discussed oily fish species, their consumption could present antioxidant benefits to some extent. 

Regarding the valorization of oily fish by-products, the heads of *P. glauca* have been used to isolate chondroitin sulfate (CS), which includes both chondroitin-4-sulfate and chondroitin-6-sulfate forms, with a very low 4S/6S ratio of below 0.2 [70]. Even though CS is present in bovine, chicken, and porcine, it is much more predominant in 4S form, in sharks, in which the presence of chondroitin-6-sulfate may reach up to 50%. This 4S/6S ratio has been demonstrated to be involved in the biological activity of CS. Lower proportions have been associated with anti-inflammatory and anabolic functions [71]. Indeed, the application of over-sulfated 4S/6S CS showed a high inhibition of tumor necrosis factor-α (TNF-α), which is not only involved in inflammatory processes of bowel diseases, but also in other autoimmune diseases such as rheumatoid arthritis or psoriasis [72]. Regarding the ulcers these illnesses may trigger, a collagen-composite gel obtained from the skin of *P. glauca* showed promising healing properties in in vivo models (rats) after 12 days of treatment [73].

### 3.2. Metabolic Diseases and Weight Control

Diseases and ailments related to metabolism comprise several disorders with interrelated organs and functions. In the case of diabetes or obesity, two key factors are oxidative status and the related inflammation. In this context, diet and personal life habits play a fundamental role. As such, the current number of obese individuals worldwide has been steadily increasing in the last decades, especially in developed countries [74]. Some of the main markers related to obesity are triglycerides, glucose, and LDL serum levels, which, if maintained at high concentrations for extended periods, can trigger white adipose tissue or liver inflammation, and so on. In this sense, fish oils, rich in EPA, DHA, and arachidonic acid (AA), have long been recognized as effective agents to reduce triglyceride blood levels; there is significant evidence of this in in vitro, in vivo, and clinical stages [11,66]. Although most beneficial properties of these ω-3 PUFAs have been attributed to fish oil consumption, some observational and prospective studies have evidenced they can also be acquired through the ingestion of fish flesh. Indeed, various recent meta-analyses have concluded that oily fish consumption can reduce serum triglycerides and increase the low-density lipoprotein (HDL) cholesterol [22]. A study based on data from Korean Health and Nutrition Surveys showed a reduction or maintenance in serum LDL cholesterol and total cholesterol levels in prediabetic individuals with a daily pattern of fish consumption of 18.0–93.0 g [23]. The effectiveness of oily fish meat intake at lowering high levels of serum triglycerides has also been confirmed, considering potential confounders (such as smoking, and other dietary habits), which hints at the beneficial effects associated with fish consumption, especially in overweight individuals [75]. Fish consumption has been strongly related to a decrease in all-cause mortality in healthy individuals [76]. These studies also unveil that these effects are generally observed with an intake of around 200 g per week, which would be roughly equivalent to two servings each week. The suggested mechanisms of ω-3 PUFAs from fish flesh to improve metabolic status have been mainly related to their antioxidant and anti-inflammatory capacities, which may contribute to the remission of metabolic disorders regarding these factors. However, reduced lipase secretion and pre-adipocyte differentiation inhibited by DHA have been suggested as possible cellular mechanisms, although these outcomes have only been reported in vitro [77]. Other mechanisms related to fish consumption point to the action of other molecules. For instance, the relatively high taurine levels in oily fish meat have also been suggested to contribute to ameliorating serum lipids and reductions in body weight [78]. Altogether, although several other factors should be accounted for (e.g., other dietary sources of ω-3 PUFAs, unhealthy dietary habits, and lifestyle choices), scientific evidence is consistent and points to the benefits associated with frequent oily fish consumption, such as lowering LDL cholesterol, increasing HDL cholesterol, and lowering plasma triglycerides, which result in a healthier metabolic status and in the prevention of developing obesity and related disorders.

### 3.3. Cardiovascular Diseases (CVDs)

CVDs are the leading cause of mortality worldwide, with a higher incidence in middle and low-income countries. WHO states that CVDs were liable for the death of 17.9 million people in 2019, representing 32% of all deaths and 38% of all premature deaths (below the age of 70) [79]. Therefore, maintaining cardiovascular health and preventing the development of CVDs is of relevance. Several factors influence cardiovascular health, including diet and lifestyle. In this sense, one of the major factors leading to the development of several CVDs is atherosclerosis, which consists of LDL cholesterol deposited in blood vessels [80]. This results in a reduced blood flux and superior cardiac effort to compensate for it. Because these deposits may also be calcified, they harden the blood vessels, which results in a loss of the vessel contractile capacity and further increases the cardiac effort and blood pressure. All of these factors may result in cardiac arrest, stroke, arrhythmias, and other related cardiac fatal events [81]. Oxidative stress and, foremost, high LDL cholesterol and triglyceride blood levels, are some of the most common exogenous factors involved in the development and onset of CVD. Therefore, reducing triglyceride and LDL cholesterol blood levels is an effective preventive treatment for CVD. The consumption of one to two servings of seafood per week and up to three to four servings per week has been associated with a lower risk of coronary heart disease mortality in adults and is compatible with the current intakes and recommendations in most countries [82,83,84]. Indeed, there is extensive evidence of such benefits for cardiovascular function provided by the intake of w-3 PUFAs and oily fish [78]. The cardioprotective effects of oily fish consumption are as a result of a reduced probability of developing arterial stiffness and atherosclerosis. However, other mechanisms induced by oily fish intake also improve cardiovascular protection. A clinical trial found that a 500 g/week intake of oily fish for four weeks significantly reduced platelet-monocyte aggregation [85]. Platelet-monocyte aggregation is a key factor in developing atherothrombosis, and this aggregation also results in inflammatory events. The more recent FiSK clinical trial performed on 8- to 9-year-old Danish children assessed the effect of substituting chicken for oily fish (~300 g/week). The study found that the treated group of children effectively improved their triglyceride, LDL, and HDL cholesterol profiles, while also reducing heart rate [86]. DHA has also been reported in a cohort study to enact cardioprotective effects besides those related to metabolic markers. The authors reported a significant reduction in arterial stiffness in metabolically healthy men, which was correlated with higher DHA blood levels [66]. All of these factors have been pointed out in recent meta-analyses. One such analysis, authored by the American Heart Association, concluded that there is robust evidence to suggest that both w-3 PUFAs supplementation and oily fish intake can be considered effective at reducing the onset of CVDs and associated deaths [87]. Therefore, it is well established that consuming seafood, especially oily fish, lowers the risk of mortality from coronary heart disease, and there is an absence of scientific evidence of the risk of coronary heart disease associated with MeHg [12].

### 3.4. Neurological Disorders 

In recent decades, it has been determined that EPA, DHA, and AA are essential components that promote neurological development and function [88]. This is due to the higher presence of ω-3 PUFAs in brain cells as part of the phospholipid cell membrane [89]. However, ω-3 PUFAs are not uniformly distributed in all cell membranes. DHA constitutes 30–40% of membrane phospholipids in the gray matter of the cerebral cortex and the retinal photoreceptor cells. DHA comprises 10–20% of the total brain lipids, whereas α-linolenic acid (ALA) and EPA combined reach less than 1% [90]. Although some ω-3 PUFAs are found in other cell membranes, only in the brain and retina do they make up such a significant proportion [11]. Because of this, it has been observed that increased ω-3 PUFAs blood levels (which can be achieved following intake) increase cell membrane fluidity and ease of neurotransmitter transportation, which can aid in improving and/or preserving cognitive function [91]. The role of oily fish ω-3 PUFAs as potential neuroprotective agents has been studied in the last decade for these reasons. Considering the expected physiological differences and needs between younger and elder individuals, the outcomes following the trials conducted are diverse. For instance, the DEPOXIN trial also analyzed if the increased intake of ω-3 PUFAs fish oil could improve depressive symptoms [92]. The authors found that following a significant increase in ω-3 PUFAs, concerning ω-6 fatty acids, there was an improvement in depressive symptoms. Regarding the effects of these ω-3 PUFAs on cognitive decline and neurodegenerative disorders, the evidence suggests that the intake of oily fish has a potential neuroprotective effect. A recent meta-analysis surveyed various clinical trials assessing whether the intake of oily fish and/or fish oil supplementation could be associated with lower incidences of dementia and Alzheimer’s disease [76]. After analyzing the data, the authors suggested that oily fish intake of up to two servings (250 g) per week could be associated with a 10% reduction in all-cause dementia and a 30% reduction in Alzheimer’s disease incidence. On the other hand, alterations in EPA or DHA levels following fish oil supplementation did not appear to improve cognitive performance. Still, they did improve executive function (especially verbal capacity and fluidity) [76]. Altogether, the intake of ω-3 PUFAs through diet has been shown to exert a potential preventive effect on the onset of neurodegenerative diseases, as well as a valuable element for neurogenesis and development. It should be noted that in its latest report on the topic, as of 2014, EFSA stated that the consumption of oily fish in one to two servings per week was recommended for pregnant women, as it could be associated with benefits in neurodevelopment, in contrast with those that did not consume it on that basis during pregnancy [93]. Arguably, the consumption of EPA, DHA, and AA through intake could also aid in the neurological development of children and adolescents, although in controlled servings and frequencies, following public health agencies’ regulations.

The potential use of fish by-products has been considered in very different scientific fields, including neurology. An experimental work analyzed the content source of glycosaminoglycans, a type of acid polysaccharide, in the fins of 11 Elasmobranchii. Among them, *I. oxyrinchus* and *P. glauca* displayed representative contents of CS/dermatan sulfate (DS); this result is relevant as highly sulfated DS has been found to exhibit both a neurite outgrowth-promoting activity and anticoagulant activity. However, as previously explained for CS, the ratio of di-sulfated disaccharides in DS is also directly involved in its biological activity. It was observed that despite the low sulfation degree of these CS/DS of a marine origin, they still stimulated neurite outgrowth of hippocampal neurons in in vivo experiments performed in mice. This neurite outgrowth-promoting activity was associated with their chemical composition, specifically their di-sulfated disaccharide ratio. When porcine CS/DS, which mainly presents a mono-sulfated disaccharide DS, was assessed, it did not show a neurite outgrowth-promoting activity. This work suggested that oligosaccharides, consisting of a cluster of di-sulfated disaccharides, such as those found in these marine species, may have the capacity to interact with the growth factors involved in neurite outgrowth [94]. Therefore, the deeper study of CS/DS from pelagic fish may open the door to developing alternative treatments for neurodegenerative diseases. 

### 3.5. Bone and Cartilage Degenerative Diseases

Regarding the use of by-products obtained from these marine species, they have been repeatedly assessed for their potential to treat degenerative illnesses or injuries. For instance, the skin of *P. glauca* was used to create tridimensional scaffolds of collagen or collagen/hyaluronic acid (20:1), which aimed to induce the chondrogenic differentiation of adipocytes. For these experiments, adipose stem cells were isolated from the subcutaneous adipose tissue of patients submitted to liposuction procedures. The success of the adipocyte differentiation into chondrocytes was mainly due to the high micropore interconnectivity of the 3D structures, which was obtained through cryogelation. The microscopical interconnection of the scaffolds triggered the adhesion and proliferation of cells but also the formation and infiltration of the extracellular matrix within the structures. The expression of several chondrogenesis-related markers (Coll II, SOX-9, ACAN, and COMP) was analyzed to demonstrate the differentiation of the adipocytes into the chondrogenic lineage. All of these markers were upregulated when the experimental conditions involved the use of collagen/hyaluronic acid structures cultured in a chondrogenic medium. Indeed, the presence of hyaluronic acid was suggested to play a crucial role in maintaining the chondrocytic phenotype at later stages. This in vitro work represents an insight into the potential use of adipocytes from liposuctions, together with collagen and hyaluronic acid, to regenerate and recover the functionality of native cartilage affected by injuries or degeneration [95]. Another example of the further application of fish sub-products is the recovery of minerals. In this case, hydroxyapatite (HA) was extracted from *X. gladius* bones following different protocols. The material treatment performed at 600 °C provided an HA containing a carbonate amount of 5%, which is like that of human bones. Besides, this HA showed the presence of minor minerals such as Na, K, Mg, and Sr, and its cytotoxicity was discarded. This calcium orthophosphate represents a promising material for biological substitutions of apatite in human bones, as, apart from being a natural biological material, they contain key minerals in bone healing processes, like Mg or Sr [96].

## 4. Importance of Consumption during Different Stages of the Life Cycle

### 4.1. Risk-Benefit Ratio: Toxicological Assessment

Several types of fish contaminants, including parasites, mycotoxins, microplastics, and heavy metals, are considered by food safety international agencies when evaluating the risk-benefit associated with the ingestion of *P. glauca*, *I. oxyrinchus,* and *X. gladius* [97,98,99]. However, one of the major causes of concern nowadays is the content of certain chemical pollutants, such as Hg and MeHg [45]. Fish consumption implies a significant dietary exposure to Hg, concentrated in fish muscles as MeHg as the predominant form; inorganic and elemental Hg is transformed into MeHg by microbes living in sediment, water, soil, and even human bodies [100,101]. Unlike elemental Hg compounds, inorganic Hg is not lipid soluble and cannot cross the blood-brain barrier [8]; however, the methylation of Hg turns into a more toxic metal owing to its easy and rapid absorption by the gastrointestinal tract. Furthermore, it may act as a neurotoxin and be harmful to different tissues and organs [14,102]. Human overdose with organic or inorganic Hg has been described to produce a variety of adverse effects in the central nervous system, including behavioral changes, tremors, headaches, hearing and cognitive loss, dysarthria, incoordination, hallucinations, and death, whereas in the cardiovascular system, this metal induces hypertension in humans and animals [8]. A significant health consequence of MeHg intake at the levels commonly found today is related to intelligence quotient (IQ) decline in children whose mothers have high levels of metal in their bodies [11]. In this regard, WHO and FAO have established a provisional tolerable weekly intake (PTWI) for MeHg of 1.3 µg/kg body weight [12]. Moreover, in Europe, consumers are protected by Commission Regulation (EC) no. 1881/2006, which sets the maximum Hg level as 1.0 mg/kg wet weight to commercialize high-level pelagic predators [103]. In this regard, most sharks and swordfish consistently complied with the EC maximum Hg levels (Table 2) and did not constitute a new risk to human health. The levels of Hg in these species from different locations are included in Table 2 and described below. 

Storelli and co-workers aimed to assess the risk-benefit balance associated with shark consumption by analyzing the Hg and MeHg levels in 15 different elasmobranch species, including *P. glauca,* purchased at an Italian market [104]. The Hg and MeHg levels found in this species were 0.63 and 0.57 µg/g, respectively, representing lower values than the average Hg levels from the other fish species analyzed in the same study (0.83 µg/g for Hg and 0.73 µg/g MeHg) [104]. In parallel, a total of 40 specimens of *P. glauca* from the north-eastern Atlantic (between the Iberian Peninsula and the Azores archipelago) caught in 2012 and 2013 were considered for total Hg analysis together with 48 specimens of *I. oxyrinchus* from the same place [105]. The results showed that the Hg concentration in *P. glauca* (0.52 mg/kg) and in *I. oxyrinchus* (0.74 mg/kg) were much lower than the EU regulatory threshold of 1.0 mg/kg wet weight. Similarly, in 2011, a total of 69 *I. oxyrinchus* and 39 *P. glauca sharks* were collected in South Pacific waters in front of the Chilean coast to determine the levels of heavy metals, including Hg [106]. The levels were 0.048 mg/kg (wet weight) for *P. glauca* and 0.034 mg/kg (wet weight) for *I. oxyrinchus* [106]. In accordance, Vellez-Alvared and colleagues measured the content of Hg in the liver, kidneys, and muscles from 20 individuals of *I. oxyrinchus* collected in Isla Magdalena, South of California, in May 2008 [107]. The results showed that none of the samples exceeded the limit level. The higher values were found in the muscles, ranging from 0.08 to 0.49 mg/kg (0.4 mg/kg on average).

In general, the bibliography showed that Hg levels in *P. glauca* and *I. oxyrinchus* were lower than in *X. gladius* (Table 2). *X. gladius* are distributed worldwide in tropical and subtropical waters and are at the top of aquatic food chains. These fish have a high rate of food intake as their metabolism is also high and, consequently, can accumulate a larger amount of heavy metals [108]. Although half of the total alerts for Hg in the EU during 2016 and 2017 were attributed to *X. gladius* [109,110], some recent studies based on specimens captured in EU waters showed that the levels complied with the legislation and were comparable to other seafood. For example, Mehouel and co-workers evaluated the content of heavy metals (including Hg) in 30 samples of *X. gladius* and 70 samples of sardine (*Sardina pilchardus*) captured in the Mediterranean Sea between May and December 2015 [111]. The results from *X. gladius* showed Hg levels below the legislated limit in all of the samples, with an average value of 0.56 mg/kg and representing a lower Hg concentration than those found in *S. pilchardus* (0.62 mg/kg). In another study, a total of 26 specimens of adult *X. gladius* were caught together with 11 other species considered to be their prey, on the Catalan coast of Spain (Mediterranean Sea) between June and August 2018 [112]. *X. gladius* showed an average Hg value of 0.66 mg/kg and, surprisingly, the Hg levels of some of the prey were higher. These species included the deep water rose shrimp, the blue whiting *Micromesistius poutassou*, and the Atlantic horse mackerel *Trachurus trachurus,* with Hg values of 0.93, 0.80, and 0.84 mg/kg, respectively. Finally, a comparative report on Hg levels in 220 commercial samples of *X. gladius* from different FAO fishing areas showed that 181 samples, representing 82.3%, did not exceed the Hg maximum limit set by EU legislation (1.0 mg/kg, wet weight), and that 85 (38.6%) were below 0.5 mg/kg [113]. Most of the remaining non-compliant samples were from the Western Indian Ocean—8 out of 21 (38.1%). Just one sample from the Mediterranean Sea (1.66 mg/kg), one from the Central East Pacific Ocean (1.10 mg/kg), and one from the Southwest Pacific Ocean (2.13 mg/kg) overpassed the limit. Furthermore, the mean Hg contamination levels observed were below the tolerable limit set by the European Community for all samples and the FAO fishing areas [113]. 

**Table 2:** Hg levels in *Prionace glauca*, *Isurus oxyrinchus,* and *Xiphias gladius* captured from different in the last 15 years. The percentage of samples below the maximum permitted level in EU legislation (MPL = 1 mg/kg) (Commission Regulation (EC) No. 1881/2006) is included [103].

Size/Weight	N	Origin	Year	Avg Hg Level (and Range) (Muscle, mg/kg, *w*/*w*)	% Samples < MPL Positive Impact Results	Ref.
***Prionace glauca***						
Large (>195 cm) Small (≤195 cm)	39	South Pacific waters (in front of Chile)	2011	0.048 ± 0.03 mg/kg	100% samples < MPL	[106]
160 ± 56 cm (74–284 cm)	40	Spanish and Portuguese long-line vessels (North-eastern Atlantic, Vigo, Spain)	2012 and 2013	0.52 ± 0.35 mg/kg (0.14–1.71 mg/kg)	90% < MPL; Lower level than *I. oxyrinchus* (Avg: 0.74 mg/kg)	[105]
***Isurus oxyrinchus***						
*-*	4	Coast of Santa Catarina State (Brazil)	2007	0.398 ± 0.290 mg/kg (0.086–0.492 mg/kg	100% < MPL	[114]
Juvenile	20	Isla Magdalena, South of California	2008	0.391 mg/kg (0.012–0.691 mg/kg)	100% < MPL; liver (0.001 µg/kg) Kidney (0.006 mg/kg); still lower values	[107].
Large (>285 cm) Small (≤285 cm)	69	South Pacific waters (in front of Chile)	2011	0.034 ± 0.023 mg/kg	100% < MPL	[106].
156 ± 36 cm (99–219 cm)	48	North-eastern Atlantic (Spain and Portugal, Vigo, Spain)	2012 and 2013	0.74 ± 0.56 mg/kg (0.12–2.57 mg/kg)	75% < MPL	[105]
***Xiphias glaudius***						
45.0–278.0 cm (Avg: 136.5 cm)	176	Galle, Mutwal (Colombo), Negombo, and Trincomallee areas of Sri Lanka	2009 (July)–2010 (March)	0.90 mg/kg (0.18–2.58 mg/kg)	68% < MPL	[115]
90–260 cm	74	Seychelles EEZ	2013 (Nov)–2014 (Dec)	0.63 ± 0.32 mg/kg	87% < MPL; Gonads still lower values (0.39 ± 0.27 mg/kg)	[116]
*-*	30	Algerian coast (Mediterranean Sea)	2015	0.56 ± 0.15 mg/kg	100% < MPL; lower levels than the other fish: *Sardina pilchardus* (Avg: 0.62 mg/kg, *n* = 70)	[111];
Adults: 102–232 cm	26	Catalan coast of Spain (north-western Mediterranean Sea)	2018 (June–Aug)	0.66 ± 0.29 mg/kg	100% < MPL	[112]
100–200 g	15	Purchased in Apulian region (Italy)	2019 (May–July)	0.64 mg/kg	100% < MPL	[117]

MPL: maximum permitted level (MPL = 1 mg/kg). N: no. of samples. Avg: average. Oct: October. Aug: August. Nov: November. Dec: December. EEZ: exclusive economic zone. *w*/*w*: wet weight.

The above-exposed data strongly prove that most oily fish specimens complied with the current regulations designed to protect consumers regarding Hg exposure. In this sense, further factors need to be evaluated to provide a full report of the risk-benefit assessment associated with these three fish species. As previously pointed out, one of the key parameters to assess the risk-benefit associated with fish ingestion is their content of Se. The recommended daily allowance (RDA) for Se has been established at 0.055 mg for adults (14–52 years, excluding pregnant and lactating women) [118]. This essential trace element acts has antioxidant and anticancer properties, and its chemical interaction with Hg may counteract the toxicity related to exposure to MeHg [15,68,117,119]. The suggested mechanism of protection against Hg toxicity relies on Se and Hg being bound to form mercury selenites, which are metabolically inert and have an extremely low solubility. Thus, Se may reduce the tissue accumulation of Hg both in fish and humans. Indeed, populations with a rich dietary intake of Se are less vulnerable to MeHg exposure. Some studies have mentioned that the major form of Se in fish species is selenate, comprising 15–36% of total selenium [68]. In this sense, recent research suggests that sodium selenite alters the abundance and proportion of the gut flora in rats, and this regulation of the intestinal flora may promote Hg detoxification in animals [100]. Nevertheless, several characteristics affect this interaction, such as Se form, Hg (elemental, inorganic, and organic), dose, and the target organ [15]. For instance, the Se potential protective effect seems to be decreased when fish grow bigger and their Hg levels increase [102]. On the contrary, other criterium to consider for the correct estimation of human exposure to MeHg is the effective absorbed percentage, as a bioavailability of 95–100% is usually assumed for risk models [120]. However, recent findings suggest that assuming these percentages of ingested Hg are absorbed into systemic circulation may be erroneous [120]. Therefore, the risk associated with Hg exposure from fish requires further evaluations regarding the amount of Se and chemical properties in order to provide more accurate risk-benefit assessments linked to their consumption [58,102].

### 4.2. Recommended Intake per Age Group

Fish consumption is part of the cultural traditions of many populations worldwide. For many people, fish is the major energy source and source of essential nutrients like ω-3 PUFAs, proteins, essential amino acids, vitamins, and minerals. In this sense, oily fish are the most relevant source of LC-ω-3-PUFAs, like EPA or DHA [8]; however, they may also represent a source of pollutants. Both beneficial and adverse effects must be assessed to provide more accurate responses to consumers. Hence, this section aims to highlight the beneficial effects of fish consumption on health outcomes in relevant groups of the population: adults, pregnant women, children, and adolescents [12]. As explained, recommendations are made to ensure the provision of key nutrients (ω-3-PUFAs, vitamin D, iodine, and Se) and based on safety considerations like the adverse effects of contaminants present in seafood, i.e., Hg. Even though most of the recommendations refer to the quantity of fish rather than the species to be consumed, some international food safety agencies have recommended limiting the consumption of certain species, including *P. glauca*, *I. oxyrinchus,* and *X. gladius*, in children and pregnant women [121]. In general terms, a normal portion of fish for adults or the general population ranges from 100 g per week to 200 g per day, but it is mostly recommended to have two servings of about 150 g each per week [93]. However, some studies highlight the need to monitor Hg and MeHg levels in some sections of the population at risk, including pregnant women, nursing women, women of childbearing age, children, and those who consume large amounts of fish.

Regarding these vulnerable groups of the population, different approaches have been observed. An assessment of the benefits of ω-3 PUFAs consumption compared with the risks of MeHg exposure in childbearing women showed a lower risk of inappropriate neurodevelopment of fetuses in women that consumed fish compared with those who did not consume fish [12]. Indeed, during pregnancy, the normal consumption established for the general population (1–2 up to 3–4 weekly servings) has been linked to better neurodevelopment in fetuses compared with a diet with no seafood [122,123]. The current recommendations for pregnant women and children highlight choosing seafood considered to be low in contaminants (e.g., trout, ocean perch, cisco, sardine, white halibut, salmon, mackerel, herring, sprats, anchovies, carp, and prawns) and avoiding swordfish, dogfish, marlin, shark, and ray. However, some studies have shown that Hg levels in these species can be minor or comparable to the data of other non-pelagic fish captured in the same place [104,111]. 

Similarly, fish consumption is beneficial for the neurodevelopment of children and adolescents. For these groups of populations, recommendations range from 100 to 300 g of fish per week, of which there should be no more than 100 g per week of large pelagic fish. For infants, fish consumption of 10 g per week from seven to nine months of age and 20 g per week thereafter is recommended. Intakes of 40 g, 50 g, and two servings of 100 g of fish per week for children aged one year, two to six years, and older than six years, respectively, are advised [93]. As previously exposed, the content of Hg in some predators has been demonstrated to be lower or at least similar to that in other non-pelagic fish belonging to the same geographical areas and annual periods [104,111] (Table 2). Therefore, to assess the benefits and risks associated with the ingestion of pelagic fish, additional parameters such as the level of Se and the percentage of absorbed Hg concerning the amount ingested, among others, have to be considered [117,120].

## 5. Conclusions and Future Perspectives

Oily fish consumption provides numerous health benefits from a nutritional and welfare point of view. However, the consumption of *P. glauca*, *I. oxyrinchus*, and *X. gladius* has been hampered by two main factors: generally reported high levels of MeHg and public health administrations’ advice to limit their consumption [124]. The bioaccumulation of MeHg in big marine predators is of concern at a global scale. Nevertheless, further studies are required in order to establish capture areas, as significant correlations have been found between the geographical regions of growing fish and MeHg or Se accumulation levels (Table 3) [113]. This is especially noteworthy for these three species as they cannot be feasibly farmed as predators at the end-of-trophic chain. Although the expected content of MeHg in these species is assumed to be significantly higher among oily fish due to their predatory nature and size in adulthood, it has been demonstrated that MeHg is highly variable. Moreover, MeHg toxicity also depends on other factors, such as the interactions with Se and how it affects its bioavailability and toxicological potential [117]. Unfortunately, this limits the accurate interpretation of the potential health benefits that these foods may yield if incorporated into diets. Studies should assess the properties and characteristics of more species as reliable data for *P. glauca*, *I. oxyrinchus*, and *X. gladius* are scarce. Although this could possibly be due to their lower demand worldwide than other oily fish species, e.g., red tuna, further rigorous studies should be performed. Yet, scientific experts generally agree that, even if consumption frequency is precautionarily reduced, the moderate consumption of oily fish could provide substantial health benefits compared with no consumption at all, even for pregnant women [93]. 

Furthermore, discarded waste produced after processing fish, like skin, bones, and cartilage, have been demonstrated to represent a promising source of bioactive molecules. Processing and exploiting all of the potentially valuable fractions of oily fish results in their capture becoming more feasible and profitable, while furthering sustainable practices [70]. Moreover, the diverse nature and composition of these non-consumed parts prompt their use as raw biomass for new materials, food and feed, and pharmaceutical applications, and as a source for new biomolecules with unexplored potential [73]. Research in this field is still ongoing and is very recent; thus, the real value of these by-products is yet to be truly defined. 

Altogether, the nutritional composition of *P. glauca*, *I. oxyrinchus*, and *X. gladius* reveals high levels of ω-3 PUFAs, with considerable quantities of DHA and EPA, which is strongly correlated with specific health benefits. The benefits of oily fish consumption are diverse and perceptible, with most scientific literature supported by regulatory agencies confirming that such benefits may be achieved from at least one to two servings per week. However, the generally reported high levels of MeHg still hampers their consumption. On the other hand, oily fish by-products may represent a source of ingredients to create new added-value products, including different solutions for alternative treatments of degenerative and chronic diseases. Therefore, future trends regarding the exploitation of *P. glauca*, *I. oxyrinchus*, and *X. gladius* will probably not only re-evaluate the hazard-benefit balance regarding their consumption and frequency, but also explore the numerous possibilities of other fractions and biomolecules that have currently not been exploited.

## Figures and Tables

**Table 3:** Geographical differences regarding Hg and Se content and their health benefit value for *Xiphias gladius*, *Prionace glauca*, and *Isurus oxyrinchus* compared with various shark species.

Sampling Area	Typology	Hg (mg/kg)	Se (mg/kg)	Se/Hg (µmol/kg)	HBVSe	Ref.
***Prionace glauca***						
Spain and Portugal	Wild	0.35	-	-	-	[105]
Azores and Ecuador		0.2	0.084	-	-	[125]
Spain		0.350	0.102	0.74	−1	[19]
***Isurus oxyrinchus***						
Taiwan	Wild	0.305	0.457	7.83	54.64	[126]
Spain and Portugal		0.56	-	-	-	[105]
Hawaii		1.81	0.32	0.46	−11.1	[127]
Korea		0.27	0.36	-	-	[128]
***Xiphias glaudius***						
Hawaii	Wild	1.07	0.39	1.16	0.1	[127]
Mediterranean area		0.64	0.44	1.8	4	[117]
EEUU		1.31	0.63	1.23	-	[129]
Portugal		0.47	0.47	3	-	[130]
Spain		0.51	0.308	1	4	[131]
Italy		0.249	0.283	1	-	[132]
Spain		0.540	0.494	2.32	13	[19]
**Shark (various species)**						
Atlantic western central	Wild	1.48	1.24	15.66	9.11	[133]
Pacific eastern central		1.65	0.86	10.87	−0.97	
Pacific western central		0.93	0.77	9.76	6.67	
Indian Ocean western		1.54	1.57	19.90	16.15	
All regions (FAO)		1.23	0.81	10.20	3.06	

HBVSe: health benefit value of selenium.
